# A realist evaluation of a physical activity participation intervention for children and youth with disabilities: what works, for whom, in what circumstances, and how?

**DOI:** 10.1186/s12887-018-1089-8

**Published:** 2018-03-15

**Authors:** C. E. Willis, S. Reid, C. Elliott, M. Rosenberg, A. Nyquist, R. Jahnsen, S. Girdler

**Affiliations:** 1 School of Sport Science, Exercise and Health, The University of Western Australia, M408 35 Stirling Hwy, Perth, WA 6008 Australia; 2 Department of Paediatric Rehabilitation, Child and Adolescent Health Service, 37-39 Hay St, Perth, WA 6008 Australia; 3 School of Occupational Therapy and Social Work, Curtin University, Kent St, Perth, WA 6102 Australia; 4 Beitostolen Healthsports Centre, Sentervegen 4, 2953 Beitostolen, Oppland Norway; 5 Department of Clinical Neurosciences for Children, Oslo University Hospital, Kirkeveien 166, 0450 Oslo, Norway

**Keywords:** Physical activity, Participation, Physical disability, Intellectual disability, Context, Mechanism, Outcome, Child, Adolescent, Parent

## Abstract

**Background:**

The need to identify strategies that facilitate involvement in physical activity for children and youth with disabilities is recognised as an urgent priority. This study aimed to describe the association between context, mechanisms and outcome(s) of a participation-focused physical activity intervention to understand what works, in what conditions, and how.

**Methods:**

This study was designed as a realist evaluation. Participant recruitment occurred through purposive and theoretical sampling of children and parents participating in the Local Environment Model intervention at Beitostolen Healthsports Centre in Norway. Ethnographic methods comprising participant observation, interviews, and focus groups were employed over 15 weeks in the field. Data analysis was completed using the context-mechanism-outcome framework of realist evaluation. Context-mechanism-outcome connections were generated empirically from the data to create a model to indicate how the program activated mechanisms within the program context, to enable participation in physical activity.

**Results:**

Thirty one children with a range of disabilities (mean age 12y 6 m (*SD* 2y 2 m); 18 males) and their parents (*n* = 44; 26 mothers and 18 fathers) participated in the study. Following data synthesis, a refined program theory comprising four context themes, five mechanisms, and six outcomes, were identified. The mechanisms (choice, fun, friends, specialised health professionals, and time) were activated in a context that was safe, social, learning-based and family-centred, to elicit outcomes across all levels of the International Classification of Functioning, Disability and Health.

**Conclusions:**

The interaction of mechanisms and context as a whole facilitated meaningful outcomes for children and youth with disabilities, and their parents. Whilst optimising participation in physical activity is a primary outcome of the Local Environment Model, the refined program theory suggests the participation-focused approach may act as a catalyst to promote a range of outcomes. Findings from this study may inform future interventions attempting to enable participation in physical activity for children and youth with disabilities.

## Background

Current approaches to rehabilitation of children with disabilities utilise the International Classification of Functioning, Disability and Health (ICF) to assess outcomes, design and evaluate interventions, and develop services and policies [1]. To reflect the growing understanding of health and functioning, changes were made to the original World Health Organisation framework to include ‘participation’ as a key element within its guidelines on delivering healthcare [1]. Participation is defined in the ICF as ‘involvement in a life situation’ and is an essential aspect of child health, development, and wellbeing [1]. All children, with and without disabilities, have a need for participation in activities and settings that provide an appropriate level of challenge, social engagement, belonging, and autonomy [2, 3]. However, a substantial body of empirical research has demonstrated that children with disabilities experience significant participation restrictions, particularly in physical activity [4]. This is alarming, as the importance of physical activity and its promotion for all children and youth is indisputable.

Whilst there is an urgent need to develop interventions that promote sustainable active living among children and youth with disabilities, there is limited understanding of mechanisms and processes that may enable participation in physical activity in this population. A recent review has proposed participation to be considered not only an outcome of rehabilitation interventions, but also a process, whereby participation as an entry point may foster a variety of outcomes for children with disabilities [5]. Accordingly, interventions attempting to optimise participation may need to consider potential diversity among outcomes and their ‘causes’, and explore interactions between attributes of the individual, participation context, and characteristics of the environment [5].

The terms ‘environment’ and ‘context’ are often used interchangeably in rehabilitation literature to refer to factors affecting a child in their surroundings. To clarify, environment is a construct denoting broad external circumstances that may be considered as enablers or barriers to functioning, participation or development [1]; the term ‘context’ refers to the setting for participation (including place, activity, people, and objects), where the person-environment interaction occurs [6]. Current developmental theories and models emphasise the importance of understanding the social context of children and the reciprocal nature of child-environment interactions [7, 8]. Similarly, two recently published reviews of leisure participation describe the central role of social contexts in creating meaningful experiences for children and youth with disabilities [9, 10]. However, there is limited exploration of other aspects of context in participation literature [11]. While the ICF posits that *contextual factors* play a significant role in determining the extent to which a person is able to participate, the framework does not explain the mechanisms through which context influences participation as an outcome.

There is a growing body of literature attempting to optimise physical activity levels in children and youth with disabilities, however few interventions have demonstrated change in a child’s participation outcomes [12]. Beitostolen Healthsports Centre (BHC) is a rehabilitation centre in Norway, seeking to enable lifelong activity and participation for people with disabilities. Adapted physical activity represents a core theoretical component of the rehabilitation program at BHC, characterised by environmental modification to facilitate participation in physical activity [13, 14]. Adapted physical activity has been described as an intersect between therapeutic and pedagogical concepts [15], reflected in the model of service at BHC whereby a rehabilitation stay is primarily a learning process [16]. Situated learning theory posits that learning is unintentional and embedded in activity, context and culture [17]. ‘Learning’ at BHC denotes involvement in activities to enable the acquisition of new skills, activity preferences, and physical activity behaviours. ‘Situated’ describes more than the specific setting in space and time; it infers that learning is a process, shaped by participation and coexistence in social contexts [17]. The BHC program theories describe a context of interaction and learning in an environment that enables children with disabilities the opportunity to participate in meaningful physical activities.

In this article, we systematically study how and why the paediatric program at BHC (the Local Environment Model, LEM) works. To identify key combinations of context and mechanisms that trigger outcomes of the LEM, our study is based on a realist evaluation perspective. Originally developed by sociologists to explore the underlying causal processes by which programs achieve their outcomes [18], realist evaluation has been applied to complex interventions in various health settings [19–21]. Realist evaluation highlights four key linked concepts for explaining and understanding programs; (i) mechanisms (what it is about programs and interventions that bring about effects), (ii) context (features of the conditions that are relevant to the operation of the program mechanisms), (iii) outcomes (the intended and unintended consequences of programs, resulting from the activation of different mechanisms in contexts), and (iv) context-mechanism-outcome configurations (models indicating how programs activate mechanisms for who and in what conditions, to elicit outcomes) [18]. While the end result of a realist evaluation is a refined set of assumptions (a refined program theory) [18], the explicit connections between concepts are not always clear [19, 22]. In this study, we wanted to uncover the association between context, mechanisms and program outcome(s), based on the perceptions and behaviours of the program participants. We aimed to define the mechanisms by which the LEM intervention may facilitate meaningful outcomes for children with disabilities and their parents. Further, this study aimed to develop a refined program theory describing the relationship between context, mechanisms and outcome(s), to identify the configuration of features that may inform future practice and policy surrounding similar interventions.

## Methods

### Design

Principles of realist evaluation [18] underpinned data collection and analysis. Data for realist evaluation is typically collected using qualitative approaches [23], and in this study an ethnographic approach was adopted. Ethnographic methods have demonstrated utility in describing the process of change during an intervention, and how and why an intervention ‘works’ [21, 24, 25]. The iterative process of continuous data collection, analysis and reflection employed in ethnography make it possible to identify mechanisms that may enable the improvement and adaptation of interventions and services [26]. In this study, the triangulation of participant observation, interviews and focus groups was utilised to determine the relationship between context, mechanism and outcome during an immersive stay at BHC [18].

### Participants

Purposive and theoretical sampling were used to select participants for this study. In the first phase of data collection, purposive sampling [27] of children and their parents participating in a stay at BHC was undertaken. Children were selected to participate in the study if they were (i) aged between 5 and 17, and (ii), participating in the LEM intervention at BHC. Parents of children were selected to participate if they were the accompanying guardian and primary caregiver of a child participating in the stay. Children and parents staying at BHC who met the inclusion criteria were first informed about the study by the Director of Paediatric Teams. Following this, all selected children and parents received their own information sheet describing the study that had been translated into Norwegian. In phase two of data collection, participants were theoretically sampled to elaborate and refine emerging categories relating to how participation in physical activity was enabled at BHC. Theoretical sampling ceased upon reaching theoretical saturation, defined as theoretical completeness in which no new properties of the categories were identified [28].

### Description of intervention

The LEM is an intervention developed by BHC dedicated to enabling physical activity participation for children with disabilities in local environments. The intervention is goal-directed and family-centred, with focus on cooperation, education, and resource capacity building in partnership with families and communities.

Collaboration with local communities occurs 1 month prior to the intervention at BHC. Representatives from the paediatric teams at the Centre travel to the community of the families coming to stay, to prepare and engage children, parents and local service providers. The main intervention is delivered at BHC, where groups of 8–10 children and their parents stay for 19 days. The children’s stay at BHC is intensive, consisting of physical, social and cultural activities, 2-5 h a day, 6 days a week. The intervention is based on the child’s goals (e.g. learning to ski), but also designed to introduce children and their families to new and different physical activities and participation experiences (e.g. rock climbing). Three children’s groups (5–17y), one young adult group (18-30y), and one adult group (>30y) stay at the Centre and participate in their specific group program simultaneously. Follow up occurs with children, families and service providers in local communities 3 months after the stay at BHC.

### Data collection

The first author (CW, independent from BHC) spent a total of 15 weeks at the Centre, undertaking data collection over two separate time periods. This covered all seasons (summer/autumn and winter/spring), accounting for any intervention-specific differences that occur (e.g. activities, equipment). The first author lived at BHC during 2014 and 2015, and participated in the daily practices of staff, children and families at the Centre. Proficiency in the Norwegian language aided in the cultural immersion of the researcher.

**Fig. 1:**
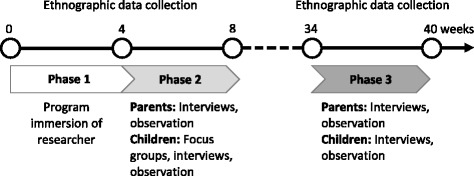
Timeline of data collection in weeks

Ethnographic fieldwork involved the triangulation of semi-structured interviews, focus groups and participant observation, employed over two time points (Fig. 1).

#### Interviews and focus groups

The first author undertook all interviews. The interviewer was a female researcher with training in qualitative data collection, with no existing relationship to the participants prior to data collection. Interview guides were developed with the assistance of a consumer-driven steering group comprised of parents of children with disabilities, an adolescent with a disability, and professionals working with disabilities in the community. The interview guides were piloted with a manager at BHC to obtain feedback of utility prior to use in data collection. The interview guides covered broad topics for discussion and were revised when new topics were raised during the interviews. Topics discussed and prompts used during the interviews with children and parents at BHC are outlined in Table 1.

**Table 1:** Key topics and prompts covered in semi-structured interview guides

Children	Parents
Participation of the child:	Participation of the child in the program
- Goals for stay	- Goals for stay
- Initial feelings about BHC	- Child’s initial feelings
- Overall experience in the program	- Describe child’s experience
Model of service:	Model of service:
- Positive and negative aspects	- Participation-related factors
- Physical activity participation	- Service-related factors
- Leisure time	- Human environment
- Human environment	- Physical environment
- Physical environment	- Similarities/differences to local community
- Similarities/differences to local community	- Recommendations
Effect on child:	Effect of stay on child
- Perceived changes (of themselves)	- Observed changes (if any)
- Recommendations for other children	
- Ongoing participation	

*BHC* Beitostolen Healthsports Centre

Semi-structured interviews (*n* = 25) and focus groups (*n* = 2) explored the mechanisms, context and outcomes of the LEM, based on the perspectives of parents and children participating in the program. Parents participated in in-depth semi-structured interviews (*n* = 18), conducted at a mutually convenient time in a private meeting room at BHC. Norway has very high proficiency in English [29], thus participants were offered the choice to conduct interviews in Norwegian (*n* = 3) or English (*n* = 15). As Norwegian was not the primary language of the first author, a translator (MM) was present during these interviews to ensure accurate interpretation of questions asked by the interviewer (CW) and answers from the interviewee. Interview duration with parents ranged from 45 to 75 min.

Two focus groups with children (*n* = 11) were conducted in phase 1, and each went for 45 min. Semi-structured interviews were conducted with an additional seven children in phases 1 and 2. Depending on the preferences of the children, these were conducted individually (*n* = 2), or with a parent present (*n* = 5). Interviews conducted individually were done so in English, and were 60 min in duration. For interviews where a parent was present, the parent acted as a translator to verify interpretations of the child’s responses by the interviewer. All parent supported interviews were 30 min in length.

The first author transcribed each interview and focus group from the recordings verbatim. Norwegian interviews were transcribed in Norwegian and translated to English by the first author. Whilst researchers who also act as translators are rare, this method enhances the validity of interpretations as it allows close attention to cross cultural meanings and understandings [30]. English translations were then back-translated by the translator that was present in the interviews (MM). Credibility was enhanced by the researcher documenting reflections in a journal following the interviews and demonstrating an audit trail of the research methods [27]. Approximately half of the interview participants had the opportunity to review their transcribed interview, and made no changes.

#### Participant observation

During phases 2 and 3, overt observational methods were used to determine relationships between viewpoints from interviews and the actual behaviours of children and parents [31]. Observations of children and parents occurred in a range of settings at BHC; throughout intake and evaluation interviews, in structured intervention activities (e.g. bike riding, swimming), and during periods of informal interactions and communications (e.g. break times). Conversational interviews with children and parents also occurred spontaneously in these settings. Observations of children and parents occurred during the hours of their typical day, 8 am-8 pm. Non-participants (i.e. individuals aged 18 years or older and/or families participating in an alternative program) were present during the observation period, and while aware of the research project being undertaken, no record of their actions, behaviours or discussions were documented.

Observations provided insights into the phenomena experienced by children and parents at BHC, and enabled the description and linking of mechanisms and outcomes identified from the interviews specific to their proposed context. Detailed field notes were documented immediately following each observation period, containing descriptions of events, conversations, reflections, ideas for further investigation, and preliminary thoughts in relation to the identified mechanisms observed in practice. This allowed exploration, reflection, and reflexive engagement to occur as an iterative process during data collection and analysis [26]. Daily contact with participants meant it was possible to check and confirm the meanings of their behaviour, and adjust or add to the field notes accordingly [32].

### Data analysis

#### Interviews and focus groups

Nvivo (QSR International Pty. Ltd., 2014) software was used for handling interview data and field notes. Discussions were transcribed verbatim and compared with field notes taken during interview and observation sessions. Transcripts were analysed using direct content analysis [33] and guided by the context-mechanism-outcome (CMO) framework used in realist evaluation. A phrase was coded as context if it described the circumstances that formed the setting for an event and/or experience. Mechanisms were components of the program that were proposed to create outcomes. A phrase was coded as an outcome if it described the impact of the program on the child [23]. After applying the CMO coding framework, data within each domain were reviewed to merge similar codes and synthesise the mechanisms, context and outcome themes of the intervention. The first author coded all interviews, and a second author (SG) reviewed and checked the coding with no disagreement.

#### Participant observation

Descriptive and thematic analysis of observation data recorded in the form of field notes occurred away from the clinical field, but onsite at BHC. This involved elaborating upon, completing and refining descriptions of fieldwork experiences, reflecting upon the emotional responses of children and parents, and examining patterns in behaviour. Observation data was coded in the same manner as the interview transcripts, to synthesise observed mechanisms, context and outcomes. Mechanisms and outcomes identified in interviews also emerged from the contextual descriptions and observed participant behaviours. The triangulation of data demonstrated comparable conclusions from each method, strengthening the internal validity of the interpretation [34].

#### Realist evaluation

The intent of realist evaluation is to develop a set of possible relationships between the context, the intervention mechanisms, and the outcomes [23]. In this study, we wanted to identify the connections participants made between the features of the context, the program elements and the outcomes they experience. In addition to individual codes assigned in the qualitative coding (a discrete C, M or O), we focused on identifying strings of CMO linkages (CO, MO, CM, CMO) within each code [23]. Generating the CMO connections empirically from the data allowed us to explore the different constellations of specific contexts and outcomes that participants themselves identified. Common links and consistent patterns between context, mechanisms, and outcomes across the data were identified to generate a context-mechanism-outcome configuration. The context-mechanism-outcome configuration is a model that indicates how the program at BHC activated mechanisms amongst children with disabilities within the program context, to enable participation in physical activity.

### Trustworthiness

All four aspects of trustworthiness were addressed to ensure the overall rigour of the research. Triangulation of data sources, prolonged engagement at the site, and persistent observation strengthened the credibility of interpretations [34]. The sampling strategies and detailed descriptions of participants (Table 2) enhanced the transferability of the data. Dependability was address by the documentation of researcher reflections and demonstrating an audit trail of the research methods [27]. Results were presented to the steering group in Australia as a method of confirmability [27].

**Table 2:** Participant demographics

Characteristic	Category	Total
Number of participants	Children (*n*)	31
	Parents (*n*)	44
Parent relationship to child	Mothers (*n*)	26
	Fathers (*n*)	18
Characteristics of children	Age, y:m (*SD*)	12:6 (2:2)
	Age range, y	6–17
	Gender (*n*)	
	Male	18
	Female	13
	Child’s primary health condition (*n*)	
	Cerebral Palsy	12
	GMFCS I/II/III/IV/V	5/4/1/1/1
	Acquired brain injury	2
	Intellectual disability ^a^	17
	Mild	5
	Moderate	12
	Number of stays at BHC (*n*)	
	1st	20
	*n* > 1	11
Semi-structured interview participants	Children (*n*)	16
	Parents (*n*)	18
	Mothers	16
	Fathers	2

*SD* standard deviation, *GMFCS* Gross Motor Function Classification System

^a^ including Down Syndrome, Fragile-X syndrome, and craniosynostosis

## Results

### Participants

All participants (*n* = 75) accepted invitations to participate in the study, and all provided informed consent (and assent). Thirty one children and their parents (*n* = 44) participated in the study. Children had a mean age of 12y 6 m (*SD* 2y 2 m) and had a range of physical and intellectual disabilities. Of the 44 parents who participated in the study, 13 were parent dyads. A total of 16 children and 18 parents participated in semi-structured interviews or focus groups. Demographic information of all participants is detailed in Table 2.

### Mechanism, context, and outcome

Data analysis revealed a clear relationship between context, mechanisms and outcomes. Context, mechanisms and outcomes were comprised of sub codes as in a typical qualitative analysis. The study generated 39 context codes, 24 mechanism codes and 27 outcome codes. Thematic analysis revealed 4 context themes, 5 mechanisms, and 6 outcomes. These categories form the sub-headings of our results below. Results focus firstly on the context that describes the conditions relevant to the operation of mechanisms; secondly, the mechanisms that were operationalised within the context and produced outcomes; and lastly, the outcomes that resulted from the mechanisms and context. Context (C), mechanism (M), and outcome (O) variables are indicated within the quotes. Quotes are accompanied by an annotation that indicates whether the quote is from a parent (perspectives did not differ between mothers and fathers) or a child (perspectives were independent of age, gender and disability type). Quotes from children are accompanied by their age, and whether they have a physical disability (PD) or intellectual disability (ID). Further examples of strings of CMO linkages can be seen in Table 3.

**Table 3:** Examples of CMO linkages within themes

Context-mechanism-outcome configuration	Sample quote
*Context*	
C1. Safe	*“He [child, 11y, ID] has problems with anxiety. He normally gets very withdrawn and stressed in new situations, at times when he doesn’t feel safe, you know. But here, I have barely seen him like that. The boys have become very good friends (M3), they do everything together. And that helps him feel safe (C1)” – parent*
C2. Learning	*“I will remind her [child, 15y, ID] of the things she has learnt here…and lead her back here (C2), to remind her that she can actually do it. That’s part of the whole thing I think. She learns what to do here so we can do it when we go home” – parent*
C3. Social	*“With the group, she [child, 16y, ID] sees that the others can do things (C2). Everybody is together, so she’s not the only one working out (C3)” – parent*
C4. Family	*“He [child, 11y, PD] doesn’t want me there [in activities] anymore (C2, C3). He feels safe here (C1), so he wants me to leave (O4)” – parent*
*Mechanism*	
M1. Choice	*“When I got here (C3), they [staff] said to me, you can choose your activities…and most of the activities I chose (M1), I have been able to try in my time here. Some of them were very difficult but they were very fun (O5)” – child, 16y, PD*
M2. Fun	*“It [horse riding] is so fun and it’s fast (O5). It’s hard, but it’s fun (M2). So I like to keep trying at it (O5)” - child, 17y, ID*
M3. Friends	*We live in a small place, and he doesn’t have many friends at home. But [child, 9y, ID] has made friends (M3) here with all the boys (C3). And so he has had so much fun (O5) – parent*
M4. Specialised health professionals	*“She [staff member] is a very special person for me and my family, because she did so much for me (M4). I am so proud of what I can do now (O1)” – child, 16y, PD*
M5. Time	*“And [children] can try many things that would be very difficult to try for the first time at home (C2). You can try to ride a horse, you can try an electric car…everything. You do not just come for one day with a lot of strangers and then have to try [the activity] immediately…there is time (M5). And maybe it’s very scary the first time and the second, but that’s ok because there is time. You have time (M5) to learn (O1, C2)” – parent*
*Outcome*	
O1. Achievement	*“So now I can do it [participation goal]! It’s very exciting and I am so happy because I never…because I could never do that before. It was the first time (O1)” – child, 16y, PD*
O2. Aspiration	*“After my last stay, I have started horse-riding at home. Now I want to do competitions (O2)” – child, 17y, ID*
O3. Friends	*“Now [at BHC] I have this friend (O3), his name is [child] and he is 16y and he has the same disability as me (C3). So we have kind of the same problems and we have the same interests. So he will come home to the same place as me. And I said if you come and visit me I will show you the football place. Because now we both love football a lot (O5)!” – child, 16, PD*
O4. Independence	*“She [child, 17y, ID] becomes more independent (O4) after the time (M5) we have spent here (C1, C2, C3). You can see the difference every time” – parent*
O5. Enjoyment in physical activity	*“I have seen him [child, 14y, ID] do everything here (C2), and now you can see that he enjoys being active and doing all of the activities (O5)” - parent*
O6. Body function and activity level outcomes	*“This is so great. His [child, 9y, PD] physiotherapist at home has been saying for ages that roller-skating would be so good for his balance (O6), but we just haven’t been able to try it. It’s so fantastic that you [staff] (M4) thought to try that here today” – parent/CW observation*

*C* context, *M* mechanism, *O* outcome, *PD* physical disability, *ID* intellectual disability, *CW* first author

#### Context

Context comprised four interrelated conditions; safe, learning, social, and family. Both children and parents described all four contextual conditions.

##### C1. Safe

This refers to the emotional safety that was necessary for a child to reveal their needs and feelings, explore new environments and experiences, and for social confidence to develop. Secure human relationships were the primary mechanism attributed to creating feelings of safety:

*“The most important thing is the people. He [child, 9y, ID] has become very attached to [staff member] (M4) and the other boys in the group (M3). It’s the people that help him feel secure and safe here (C1)” – parent*

This safe context was a setting children felt they could explore their limits, take on challenges, and try new things. For children, feeling safe provided them a freedom to take risks and make errors, without the fear or need for self-protection of potential social consequences. Feeling safe facilitated learning:

*“I feel like I can try new things because I feel safe here (C1)” – child, 17y, ID*

##### C2. Learning

Learning describes a context that enabled children to acquire new (or reinforce existing) skills, behaviours and preferences, and to master new understandings. This context was shaped by the range of novel activities that constitute the intervention, and was a large contributor to a child’s engagement in the program.

*“I have learnt to try new things (C2)…Here, everyone can find something they love to do (M2)” – child, 9y, PD*

The context of learning referred not only to activity exposure and acquisition, but also to knowledge gained from being around others. Children described how *‘meeting new people and seeing people with different disabilities’* meant they *‘learnt a lot about new things’*. Parents explained that learning in a social context was important for their children:

*“I think it’s really important that our kids learn to think about others (C2). That they are not the only one to be taken care of, that others also need to be heard and that sometimes they have to wait… To see that there are other people with other needs (C3)” – parent*

##### C3. Social

The social context refers to other individuals with disabilities that children interact with throughout the duration of the program. Children described this as a place where you could *‘make friends, and just be together’*. Being together in a social group was often described by parents as *‘the best part’* of the program for their children. This was considered a motivational tool for engaging children in physical activity, particularly for children with intellectual disabilities where *‘everyone is motivating each other’*. For others, this context was meaningful just for *‘the opportunity to be around other people’*. Parents frequently described the social context as an uplifting change to the loneliness and isolation that children experienced in other social settings:

*“The kids in the street, they don’t want to play with her. She’s different, and she’s slower, and she can’t do what they do. You see how much [child, 12y, PD] just fits in here (C3)…she absolutely loves it. She wants to stay for another four weeks!” – parent*

Being around people with disabilities fostered self-reflection in children of all ages, with many describing this context as a place where a child *‘felt like I could be myself’*. Some parents felt this was a learning experience that would shape their children’s lives:

*“When we came, [child, 9y, PD] said, ‘What am I doing here? There are so many people that are different (C3)’. And we had to have a talk about being different. Before, she thought that she wouldn’t have cerebral palsy when she grows up. And now she understands (C2), ‘maybe I will have [cerebral palsy] my whole life’ ” – parent*

##### C4. Family

Family, notably primary caregivers, were also considered in the circumstances that form the context of the program. Initially, children were happy to explore the new environment (BHC) as long as they were in the presence of secure attachment (caregiver). Children became anxious in the presence of novelty (e.g. activity) when their caregiver was absent:

*‘The first activity this morning was ‘activity bingo’. This wasn’t an activity that parents were invited to participate in. However, [child, 11y, ID] refused to let go of his Mum’s hand (C4). [Child’s Mum] stayed with us for the warm up, but was firm saying she would not join [the activity]. [Child] looked absolutely terrified, but [staff member] (M4) convinced him to join him and [friend] in the activity (C2)’ – CW observation*

As relationships between staff and children developed in the engaging environments, children’s sense of security deepened. For younger children, participating without the presence of parents often was a novel experience, one they were proud of, generating a new sense of independence they wanted to further explore:

*“Now I can stay without Mum (C4) in the swimming pool, and in the big hall and in the small gym and on the horse (O1, O4). And today is the first time Mum won’t be with me for the push bikes” – child, 9y, PD*

Children generally enjoyed having their parent(s) with them during the program, describing the experience as *“very fun” (O3).* Only one child (male, 15, ID) disagreed, describing his mother as *“embarrassing”.*

#### Mechanisms

Five mechanisms were identified by children and parents.

#### Child identified

One mechanism was identified solely by children as an important factor for inducing program outcomes.

##### M1. Choice

Choice was identified by children as a mechanism that facilitated engagement and enjoyment in physical activity, and aspirations for future participation. While a child’s program at BHC is based on their participation goals, they are exposed to a variety of physical activities. Choice and voice during goal setting, within the activity program, and outside of formal activities, was an essential element for a child’s engagement and enjoyment. As one adolescent girl described,

*“I have been swimming a lot and I went to the disco! (C2). But I don’t do shooting. I do some of the activities but only the ones I want to (M1)” – child, 16y, ID*

This experience of both choice and variety was helpful for some children in exploring their activity preferences. The operationalisation of choice in the learning context encouraged children to consider their ongoing participation in physical activity and future participation opportunities.

*“We have tried different things here (C2), so we have more to choose from when we go home (M1). Now I have ideas of the things I want to do when I go home (O2)” – child, 17y, ID*

#### Child and parent identified

Three mechanisms were identified by both children and parents as factors that induced program outcomes.

##### M2. Fun

Fun was identified by children as a mechanism that created enjoyment in physical activity, and motivated children to achieve their goals. If the activity was not fun, this outcome was not achieved. Children often explained this in relation to both the learning and social context:

*“I hate swimming at school. It’s not something I love. But the swimming here with everyone (C2, C3) is so fun. At school it is boring” – child, 9y, PD*

Parents believed in the inherent value of activities being fun, a mechanism essential for motivation and progression. Parents frequently described fun as a covert mechanism to achieving body function-based outcomes that were meaningful to them:

*“Rock climbing is so good for his [child, 6y, PD] arms. It’s strengthening his arms a lot and it’s a good way of building his self-confidence because he will manage to climb different kinds of routes (O1, O6). So it’s not only fun (M2), it’s good for him also (O6). Like all of the activities here.”*

##### M3. Friends

Having friends was a unique variable, where it was identified by parents and children as both a mechanism and an outcome. Both parents and children described friends as the reason for such positive experiences in the program. These were often so meaningful that children aspired for these relationships and positive experiences to be a permanent part of their future:

*“The best would be to live with my friends [from BHC] (M3) with all of our happy dogs and be happy all together (O2)” – child, 16y, ID*

Friends were a salient feature of outcomes of achievement and enjoyment in physical activity. They provided motivation that enabled children to persevere when activities were *‘hard’* or *‘uncomfortable’*. Sharing these achievements with their friends was also highly meaningful to children:

*‘He [child, 14y, PD] was the last to finish the cycle course, and all of his friends were cheering him on, helping him to finish (M3). When he crossed the finish line, he had the biggest smile on his face. He was so proud, and so thrilled to see that everyone was cheering for him. He punched two hands in the air, threw his head back, and said ‘yes’! (O1) – CW Observation*

##### M4. Specialised health professionals

Health professionals were a mechanism that influenced all outcomes. Children described staff as *‘the world’s best’*, explaining the crucial role of staff in enabling goal attainment, and performing and participating in activities independently:

*“Because the people who work here (M4), they help you and tell you how you can do it on your own (O4)! So it makes it very easy and very fun to do things here (O5)” – child, 16y, PD*

The abilities of the health professionals to adapt physical activities to the needs of each individual did not go unnoticed by children or parents. For parents, having specialised staff *‘is so important’* and made it *‘easier to let go’* during the program. Parents perceived staff as providing a highly individualised model of service, contributing to creating a safe learning environment:

*“Here, the whole team (M4) work together and everyone knows my daughter. They know when to push [child, 16y, ID] and they know how to motivate her to try new things (C2). Often, she really wants to do [an activity] but she is scared. But the staff here keep trying and break things down into small steps. So it’s a safe place to do things (C1), because the staff are genuinely interested in the child and they know the child so well. They try and try and try, with whatever each child needs. They are fantastic. Nothing is a problem for them” - parent*

#### Parent identified

One mechanism was identified only by parents as being a factor that induced program outcomes.

##### M5. Time

Time was discussed by parents as mechanism that facilitated the evolvement of context. Time was what children needed to ‘*feel secure and to feel safe’.* Time facilitated learning, and allowed children to attempt activities at their own pace. Time was a requirement for group development, which formed the basis for peer relationships:

*“For her [child, 12y, PD], making friends (O3) is something that takes time (M5)” – parent*

Time was an important mechanism for all outcomes, ‘*important because it means the children do not feel stressed with change’.* Time was discussed in relation to changes in body function, as a mechanism that enabled children ‘*to focus on how to use their bodies’*. Time was described as crucial for mastery of skills, achievement of goals and independence in social and activity settings. Importantly, time enabled children to enjoy the participation experience:

*“She [child] is really afraid of horses. But now for the first time, they have been talking to the horses every day. And the other day, she was sitting on a horse. And she was so proud (O1). Smiling and laughing and waving (O5)! But my goodness, before she was so afraid. And now [with time] (M5), she is perfectly fine” – parent*

#### Outcomes

Six program outcomes were identified by children and parents:

#### Child identified

Two program outcomes were identified solely by children.

##### O1. Achievement

Achievement refers to the mastery experiences that children experienced during the program, an outcome that resulted from the attainment of participation goals, or successful attempts at novel activities. Achievement was a highly meaningful outcome for children:

*“It [achieving participation goal] is such a big thing for me. I cannot tell you how much in words. It’s so big, I cannot tell you how big it is” – child, 16y, PD*

##### O2. Aspiration

Aspiration describes the ambition that children acquired during the program. Children were able to recognise and understand their capabilities, which encouraged them to consider goals for the future. Aspirations related to building on their physical activity participation achievements:

*“Now I want to learn how to balance [on the bike] by myself” – child, 9y, PD*

Some children looked further into the future, and applied their skills and participation experiences to employment aspirations:

*“When I grow up, I want to be a professional footballer” – child, 10y, PD*

#### Child and parent identified

Three program outcomes were identified by both children and parents.

##### O3. Friends

Friendships were perceived as a highly meaningful outcome of the program for both children and parents. Children typically explained the outcome of friends as a quantity, i.e. *‘now I have many friends’*, and that *‘the best part [of BHC] was making my first friends’*. The significance of these (new and growing) friendships was reinforced by parents, particularly for those whose children had participated in multiple stays at the Centre:

*“I think that the best thing out of it the first time was all of those friendships. And that they have stayed together ever since” – parent*

The data revealed that context facilitated friendship development, rather than specific mechanisms. Children and parents described friends as an outcome of the safe, social context:

*“[At home] he [child, 11y, ID] has no close friends. Just because he is different. Here, he feels safe (C1). He is close to everybody (C3). The boys are a ‘pack’!” – parent*

##### O4. Independence

Parents described independence as an outcome that occurred as a result of the time spent in the context of the program. Independence in physical activity was an important facilitator to a child’s ongoing participation:

*“When we take a bike trip with my kids, I always have to stop and help him [child, 14y, PD]. And his bike, it’s so heavy with all of its chairs and wheels, and I have to help both his and my bike over the road. But now he’s able to do it himself” – parent*

Children described this outcome in terms of being able to manage skills and activity participation without the assistance of others:

*“I am more independent. I get help if I need it, but now I can do it myself” – child, 11y, ID*

##### O5. Enjoyment in physical activity

Parents described their initial desires in the program for their child to *‘enjoy being active’* and *‘feel motivated to participate in physical activity’*. Parents wholeheartedly believed that BHC had enabled positive physical activity participation experiences for their child. This was generally described in relation to context:

*“…we were up in the mountain. That was so much fun (O5)! And when we got there, I forgot that she [child, 16y, ID] was so afraid of snow. She is afraid of just walking in the snow. She always cries. And this time, after having tried the snowshoes [at BHC] (C2), she was fine. Absolutely no problem. She forgot that she was afraid of the snow and enjoyed the walk (O5)” – parent*

Children described physical activity participation at BHC as highly enjoyable and *‘very fun’*, with no children inferring any negative feelings towards their experience. Children *‘would like to stay for longer’*, and if given the opportunity, would let other children know that:

*“When you come here, you just have fun. That is very important. And you are active. They are the two most important things to know. Being active is fun.” – child, 16y, PD.*

#### Parent identified

One program outcome was identified only by parents:

##### O6. Body function and activity-related improvements

While not the primary motivation for participating in the program, parents expressed the importance of the health and functional benefits gained from the physical activities at BHC. In addition to perceived physiological changes, those related to a child’s personal disposition were an outcome observed by most parents:

*“She [child, 16y, ID] has really benefitted from all of the physical activity. She can last longer in activities, and she is happier and more confident in herself and what she can do (O6)” – parent*

A child’s skill development, both activity-based and in a social context, was a meaningful outcome for parents. Parents not only observed these improvements, but also commented on how quickly they were attained:

*“In her [child, 12y, PD] social confidence, in her balance…at home this is rare but here it’s happened so quickly. It’s amazing how easy it comes. So quick. And I thought that this is not going to happen, but it did, and so quick!” – parent*

Children did not make reference to perceived health benefits. However, one adolescent (GMFCS IV) commented on his participation in a gym program:

*“When I came here I trained to be a small boy, and not be a fat boy. But when I was here and I talked to people (C3), they helped me to understand that it’s not good to think in this way. It’s good to think that when you train, you will be stronger (O6). And you will be able to help your father and you will be able to help yourself (O4)” – child, 16y, PD*

#### Context-mechanism-outcome configuration

**Fig. 2:**
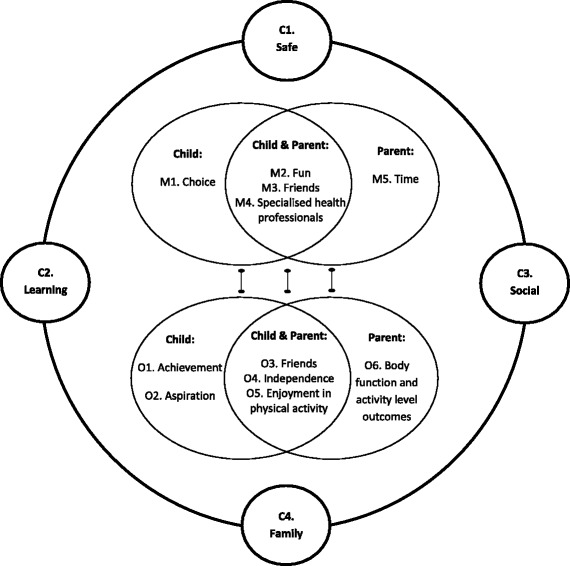
Context-Mechanism-Outcome configuration of the Local Environment Model, illustrating the mechanisms (M) activated within interdependent contexts (C) to enable physical activity participation and associated outcomes (O) for children with disabilities

As the raw data within the thematic results infer, mechanisms, context and outcomes were interrelated, as described by children, parents and the researcher (Table 3). This led to the development of a context-mechanism-outcome configuration from the LEM (Fig. 2). This generative causality model provides an account of why the outcomes transpired as they did. Thus, the causal explanation is not a matter of a singular mechanism (M), or a combination of mechanisms (M1.M2) asserting influence on an outcome (O). Rather it is the association as a whole that is explained. Accordingly, Fig. 2 removes the causal arrow and replaces it with the dumbbell shape, representing the tie or link between the set of variables (mechanism and outcome, within context), explaining the consistency between context, mechanisms and outcomes [18].

## Discussion

This study demonstrated a clear and consistent relationship between context, mechanisms and outcomes of the LEM intervention to generate a CMO model and refined program theory. This theory integrates mechanisms and context to predict and explain outcome patterns for children and youth with disabilities during a participation-focused physical activity intervention. This study identified five mechanisms (choice, fun, friends, specialised health professionals, and time) that facilitated meaningful outcomes for children with disabilities and their parents. We demonstrate that the LEM intervention activated these mechanisms amongst children with disabilities in a context that was safe, social, learning-based and family-centred, to elicit outcomes across all levels of the ICF. Of importance, this theory describes that it is not a matter of a singular mechanism (or even a combination of mechanisms) asserting influence on one outcome, but the interaction of mechanisms and context as a whole that facilitates outcomes.

Whilst optimising participation in physical activity is a primary outcome of the LEM [35], findings of this study demonstrate that outcomes for children and parents extended beyond this. Mastery experiences, independence in participation, authentic relationships, and hopes for the future were all identified by children as equally meaningful outcomes of the program. Furthermore, parents perceived the participation-based intervention to elicit outcomes at the level of body functions and activity. This is a novel finding in paediatric disability literature, with implications for rehabilitation interventions attempting to improve outcomes at these levels. This raises the question; Can participation-focused interventions, that are highly engaging for children over sustained time periods [36], contribute to improvements in impairments and activity limitations? Emerging evidence suggests that long-term involvement in exercise may improve neuromuscular characteristics and functional capacity of people with cerebral palsy [37]. In typically developing children, those who regularly participated in sport over three years displayed better motor outcomes than children who only partially participated, or did not participate in sport at all [38]. In these studies (and ours), participants were not involved in interventions specifically designed to enhance body function or activity outcomes; rather, they were participating in physical activity and exercise pursuits that were intrinsically motivating to them. The outcomes of our exploratory research warrants further investigation of this hypothesis for children with disabilities.

Our findings support the notion that effective interventions are dependent on contextual interdependencies. The interrelatedness of safe, social, learning and family contexts was required for the operation of the program mechanisms. While the role of family and social contexts in enabling participation is increasingly being documented [10], the concept of ‘safe’ learning contexts is relatively unexplored. While many articles centre on environments or strategies that ensure physical safety (of which is of utmost importance) [39, 40], our results explore the idea of a central context that is perceived by children and parents to be emotionally safe. In this study, secure human relationships were the primary mechanism attributed to creating feelings of safety. Initially, this security was provided by a child’s family, which facilitated a child’s early engagement in the learning context. As a child’s sense of security deepened, facilitated by developing relationships with staff and other children, the attachment to caregivers dissipated and children actively engaged in the learning and social contexts without fear or need for self-protection. In sociology, this experience of emotional safety is termed ‘membership’, proposed to be created by being a member of an integrated group that has boundaries and emotional security [41]. The results of this study suggest that affirming membership of a group supported a child’s inclusion through shared and emotional connections. Reconstructing ‘safe’ contexts, through the inclusion of parents and other children with disabilities within program design, may be a primary consideration for interventions aiming to engage children in physical activity pursuits. Furthermore, the family and social contexts that facilitate the development of secure relationships may assist in sustaining participation in physical activity after the intervention has ceased [42].

Five mechanisms were operationalised by the safe, social, learning, and family context of the LEM. While choice, friends, and fun have previously been proposed as mechanisms that may facilitate participation in physical activity [10], there is limited understanding of the role of health professionals in achieving this outcome. In this study, having access to specialised health professionals that provided an individualised service and were highly competent in facilitating physical activity participation was described by both children and parents as crucial to the outcomes of the program, and beyond. Despite the multidisciplinary nature of the teams, behaviours of health professionals at BHC strongly aligned with the values of adapted physical activity specialists; i.e., an abilities-based approach to practice where the focus is on the person in a learning situation (rather than in a treatment situation) [43], adopting person-centeredness, operating with openness (enabling inclusion in all types of physical activity opportunities), creating compatibility (the interaction between the person, environment, activity and participation) [44], and fostering empowerment and self-determination of individuals [13]. Results from this study not only support the importance of health professionals as mechanisms for intervention effectiveness [45], but highlight the central role of skilled professionals in enabling a child’s participation in physical activity. Health professionals working in more conventional settings can exemplify these abilities-based behaviours by actively seeking opportunities to create mutual partnerships with providers of physical activity programs, uniting families and community members who have similar experiences to facilitate support and empowerment, and engaging children with disabilities and their parents in the development of resources, adaptations, and recommendations for program design [42, 46].

### Implications for policy and practice

Outcomes of this realist evaluation offer particular advantages for practice and policy. As realist approaches acknowledge and accommodate the ‘messiness’ of real-world interventions by asking different questions (not just ‘whether’ but ‘how’ and ‘for whom’), they can inform the tailoring of interventions and policy to particular purposes (such as optimising participation in physical activity), to specific target groups, and in particular sets of circumstances [18]. This is especially relevant when considering ‘time’, a mechanism identified by parents that both facilitated the evolvement of context, and elicited outcomes. For both families and clinicians, time is a resource when it is available; and its absence often redefines time as a constraint. While intensive intervention models (whereby time is also an active ingredient) have demonstrated effectiveness in improving clinical outcomes in a research setting [47, 48], a number of barriers exist surrounding their implementation into clinical practice. Results from this study suggest that an intervention model that incorporates ‘time’ as an active ingredient may be effective for improving participation outcomes for children with disabilities; not necessarily because it allows for greater intervention dosage, but for its role in creating safe contexts, facilitating learning, and fostering peer relationships, which in turn lead to outcomes. These results may be of particular value for policy makers and funding bodies; an explanation of how a program mechanism works to elicit outcomes that are of importance for children, families, and professionals may advance the implementation of research into policy.

An important principle of realism research is that, in contrast to other research paradigms, the ‘causes’ of outcomes are not simple nor deterministic [49]. Practitioners should therefore be aware that the mechanisms of choice, fun, friends, specialised staff and time, will not cause the perceived outcomes, but they may make these outcomes more likely, if operationalised in the right contexts. Interestingly, the findings of this research were independent of age, gender, and disability of children, which may encourage its application across a range of clinical settings. The mechanisms, contexts and outcomes described in this study incorporate the essence of ‘the F-words in childhood disability’ (function, fitness, friends, family, fun and future) [50], and present a novel approach to how this widely adopted framework is incorporated into clinical practice. In highlighting the bidirectional nature of the ICF, these authors similarly encourage the reader to imagine how a child’s participation and engagement in physical activity may have an important impact on outcomes across all domains [5, 50]. While realist evaluation attempts to pinpoint the configuration of features needed to replicate an intervention or program, we encourage the application of findings from this study to be considered in parallel with broader frameworks to enhance its transferability.

### Limitations

There are a number of limitations with this research. Ethnography is a time intensive methodology, and not all interviews were able to be transcribed in the time that parents and children were at the Centre. As such, we were not able to include a complete member checking process, although 50% of participants were reached. Additionally, there were a greater number of female caregivers involved in the study, which may have impacted our understanding of the program with limited perspectives from male caregivers. Of note, this sample was reflective of the demographics of caregivers staying at BHC, as were those of the children. Finally, the results of a realist evaluation are conditioned by the nature of the programs they investigate, meaning that these findings are provisional. Whilst we have developed a theory about what works, for whom, and in what context, we encourage future investigation and testing of our hypotheses.

## Conclusions

This study provides new knowledge of mechanisms and contexts that may enable participation in physical activity for children and youth with disabilities. Understanding interactions between how a program elicits outcomes, and in what conditions, is critical to the tailoring and implementation of effective interventions. Whilst physical activity participation is a primary outcome of the LEM, the refined program theory suggests participation may also act as a catalyst to promote meaningful outcomes across all levels of the ICF. Outcomes of this study may be of particular value for policy makers, researchers, and health professionals, for further testing and utilisation in the development of interventions across paediatric disability.

## Abbreviations

BHC: Beitostolen Healthsports Centre
CMO: Context-Mechanism-Outcome
GMFCS: Gross Motor Function Classification System
ICF: International Classification of Functioning, Disability and Health
ID: Intellectual disability
LEM: Local Environment Model
PD: Physical disability
SD: Standard deviation
